# Astragaloside IV Ameliorates Myocardial Infarction Induced Apoptosis and Restores Cardiac Function

**DOI:** 10.3389/fcell.2021.671255

**Published:** 2021-07-29

**Authors:** Chuang Sun, Guangwei Zeng, Tingting Wang, He Ren, Huixian An, Cheng Lian, Jing Liu, Li Guo, Wei Li

**Affiliations:** Department of Cardiology, Xi’an International Medical Center Hospital, Xi’an, China

**Keywords:** astragaloside IV, diabetes, apoptosis, MAPK, MI

## Abstract

**Background:**

Type 2 diabetes mellitus increases the risk of cardiovascular disease including myocardial infarction (MI). Inflammation and apoptosis have been implicated in the pathophysiology of MI. In the present study, the effects of astragaloside IV (AS-IV) on MI in diabetic mice were evaluated.

**Methods:**

High glucose/high fat (HG/HF) and hypoxia culture condition were established to mimic diabetic condition. After administration of AS-IV to H9c2 myocytes, the cell apoptosis, viability, and activation of mitogen-activated protein kinase (MAPK) signaling pathways were detected. MI was induced in streptozotocin-induced diabetic mice. After administration of AS-IV to mice, cardiac function, cardiac fibrosis, inflammation, and activation of MAPK signaling pathway were detected.

**Results:**

Astragaloside IV treatment significantly inhibited HG/HF and hypoxia-induced apoptosis of H9c2. AS-IV inhibited activation of JNK and p38 signaling pathway while promoting the activation of EKR signaling pathway. AS-IV treatment rescued cardiac function, suppressed cardiac fibrosis and inflammation, and differently regulated the activation of MAPK signaling pathways.

**Conclusion:**

Astragaloside IV prevented apoptosis and restored cardiac function in MI, which may be due to the regulation of MAPK signaling pathway in diabetes.

## Introduction

Type 2 diabetes mellitus (T2DM) is a metabolic disorder with high blood sugar (DeFronzo et al., 2015). T2DM is one of the major health intimidations and causes great economic burden over the world. T2DM results in multiple clinical complications including diabetic cardiomyopathy, retinopathy, nephropathy, and neuropathy (Zheng et al., 2018).

Myocardial infarction (MI) is a common cardiac disease caused by decreased or stopped blood flow to the heart (Anderson and Morrow, 2017). The correlation of T2DM and MI has been reported. Compared with patients without diabetes, patients with T2DM have greater mortality and morbidity during MI and post infarction (Jacoby and Nesto, 1992). However, the precise relationship between MI and diabetes needs further elucidation (Lu et al., 2020).

Apoptosis has been implicated in MI. It has been detected that in the hearts of MI patients, the myocytes undergo apoptosis (Saraste et al., 1997). Extensive experimental studies also suggest that MI is with inflammation, which contributes to the pathogenesis of MI (Huang and Frangogiannis, 2018). Anti-apoptotic and anti-inflammatory approaches have been described to protect against MI (Jian et al., 2016; Shin et al., 2019).

The mitogen-activated protein kinase (MAPK) family includes three kinases: extracellular signal-regulated kinases (ERKs), the c-Jun NH2-terminal kinases (JNKs), and the p38 enzymes (p38 MAPKs). The MAPKs signaling pathways regulate various types of cellular functions including apoptosis, cell proliferation, and inflammation (Johnson and Lapadat, 2002). Activation of the MAPK signaling pathways has been detected in both ischemic and diabetic heart diseases. The MAPK signaling pathway has been described to be involved in apoptosis. For example, activation of p38 induces apoptosis in neonatal rat cardiomyocytes after hypoxia, while inhibition of p38 protects cardiomyocytes (Mackay and Mochly-Rosen, 1999). JNK also demonstrates pro-apoptotic activity, while suppressing JNK prevents apoptosis in cardiomyocytes (Mizukami et al., 2001). Therefore, inhibition of p38 and JNK signaling pathways has been shown to protect against diabetic cardiomyopathy (Qi et al., 2009; Pan et al., 2014).

*Astragalus membranaceus* is a traditional Chinese herbal medicine, which is thought to promote health. Astragaloside IV (AS-IV) is the major active ingredient in *Astragalus membranaceus* with multiple activities including anti-tumor (Cheng et al., 2014), anti-inflammation (Zhang and Frei, 2015), anti-oxidation (Gui et al., 2013), anti-diabetes (Lv et al., 2010), and anti-cardiovascular diseases (Han et al., 2011). Interestingly, AS-IV has been described to regulate the MAPK signaling pathway, suggesting its potential effects on MI. In the present study, the effects of AS-IV on MI were evaluated, and the underlying mechanisms were explored.

## Materials and Methods

### Cell Culture and Treatment

H9c2 myocytes [obtained from the American Type Culture Collection (ATCC)] were cultured in DMEM supplemented with 10% heat-inactivated fetal bovine serum (FBS, Gibco), 100 U/ml penicillin, and 100 mg/ml streptomycin (Thermo Fisher, Waltham, MA, United States) in an incubator with 5% CO_2_ at 37°C. To mimic the diabetic condition, H9c2 cells were cultured with high glucose and high-fat medium containing 33 mM glucose and saturated FFA palmitate (16:0, 500 μM) for 12 h as described previously (Dyntar et al., 2001). To induce hypoxia, cells were cultured in atmosphere with 5% CO_2_, 95% N_2_ humidified atmosphere, yielding less than 1% O_2_ concentrations for 2 h. Then H9c2 cells were treated with 10 or 50 ng/ml astragaloside IV (AS-IV) for 48 h. Phosphate-buffered saline (PBS) was used as the control. HG/HF medium was maintained during 48 h of AS-IV treatment. After AS-IV treatment, cells were harvested for analysis.

### Flow Cytometry

After treatment, single-cell suspensions of H9c2 cells were prepared and stained using FITC Annexin V Apoptosis Detection Kit with PI (BioLegend, San Diego, CA, United States). Briefly, cells were washed with BioLegend’s Cell staining buffer and re-suspended in Annexin V binding buffer. FITC Annexin V and propidium iodide (PI) were added to the cell suspension and incubated for 15 min at room temperature. After washing, the cells were analyzed in BD Facscalibur flow cytometry. FlowJo software was used for data analysis.

### Fluorescence Assay

After treatment, H9c2 cells were subjected to staining using LIVE/DEAD^*TM*^ Viability/Cytotoxicity Kit (Thermo Fisher, United States). Briefly, cells were stained with combined LIVE/DEAD^®^ assay reagents for 3,045 min at room temperature and then analyzed using a Nikon Eclipse Ti2 fluorescence microscope.

### Cell Viability Assay

Cell viability was measured using CCK-8 assay. Briefly, 10^4^ H9c2 cells were seeded in each well of 96-well plates. After treatment, CCK-8 reagent (Sigma, St. Louis, MO, United States) was added to each well at a final concentration of 100 μl/ml for 2 h. The absorbance at 450 nm was read using a microplate reader.

### Western Blot

Total protein from H9c2 cells were extracted using RIPA lysis buffer (Abcam, Beijing, China). Total protein from heart tissues were homogenized in RIPA lysis buffer. Then proteins were loaded in SDS-PAGE gel and then transferred to a nitrocellulose membrane (Abcam, Beijing, China). After blocking in 5% non-fat milk, primary antibodies were added the membrane for incubation overnight at 4°C. The next day, after washing, the membranes were incubated with relevant HRP-conjugated secondary antibodies for 1 h at room temperature. ECL Substrate Kit (Abcam, China) was used to detect immune-reactive bands. The band intensity was analyzed using ImageJ. Primary antibodies used in the present study include anti-p38 antibody (Abcam, United States, Cat # ab195049), anti-phospho-p38 (Abcam, Cat # ab4822), anti-JNK1/2 (Abcam, Cat # ab112501), anti-phospho-JNK1/2 (Abcam, Cat # ab4821), anti-ERK1/2 (Abcam, Cat # ab214036), anti-phospho-ERK1/2 (Abcam, Cat # ab21436), anti-GAPDH (Abcam, Cat # ab9485), and goat anti-rabbit IgG H&L (HRP) (Abcam, Cat # ab205718). The dilutions of primary antibody and secondary antibody were 1:2,000 and 1:5,000, respectively. The densities of bands were analyzed using ImageJ.

### Mice Treatment

The 8-weeks-old male C57BL/6 mice were purchased from Shanghai Laboratory Animal Center (Shanghai, China). Diabetes was induced by injecting streptozotocin (STZ). Streptozotocin (Sigma, St. Louis, United States) was dissolved in 0.1 M of citrate buffer, pH 4.4. To induce diabetes, mice were fed with a high-fat diet for 4 weeks and then injected with 50 mg/kg/day of streptozotocin for two consecutive days. Then mice were orally administrated with AS-IV (10 or 50 mg/kg/day) for 2 weeks. PBS was used as control. During AS-IV treatment, mice were continually fed with the high-fat diet. Animal studies were approved by the ethics committee of Xi’an International Medical Center Hospital.

### Blood Glucose Level

Fasting serum glucose level was measured using the Accu-Chek^®^ Inform II system blood glucometer (Roche Diagnostics, Indianapolis, IN, United States). Mice with fasting plasma glucose (FPG) more than 11.1 mmol/L were considered with T2DM (Yao et al., 2018).

### Myocardial Infarction Model

Myocardial infarction model was established as previously described (Gao and Koch, 2013; Lu et al., 2020). Briefly, mice were anesthetized and placed on a heating pad. A tiny cut was made on the left chest, and a small hole was made in the fourth intercostal space. When the ligation was completed, the heart was placed back. After the stature of the muscles and skin, the mice were put in an incubator for recovery until fully ambulatory.

### Echocardiography

A Vevo 2100 high-resolution imaging (FUJIFILM VisualSonics, Inc., Toronto, ON, Canada) system was used to perform echocardiographic measurements. Left ventricular ejection fraction (LVEF) and left ventricular fractional shortening (LVFS) were measured, and the data were analyzed using the VevoStrain software.

### Histology

Hearts from mice were isolated, fixed in 4% polyformaldehyde, and embedded in paraffin. Then the blocks were cut into 5-μm-thick sections. To directly visualize collagen fibers, trichrome staining was performed using the Masson’s Trichrome Staining kit (Sigma, St. Louis, MO, United States) following the protocols of the manufacturer. The viable myocardium was stained in red, and collagen fibers were stained in blue. ImageJ was used to quantitate the area of fibrosis.

### RT-PCR

Total RNA from heart tissues was extracted by RNeasy Mini Kit (Qiagen, Germantown, MD, United States). cDNA was synthesized using the PrimeScript^*TM*^ RT Reagent Kit (Takara, Beijing, China). The TB Green^®^ Advantage^®^ qPCR Premix (Takara, China) was used to set up the real–time PCR, and the real-time PCR was performed in 7,500 fast Real-time PCR System (Applied Biosystems, United States). Primers used in the present study were described previously (Meng et al., 2019). The primers sequences were *IL-1*β forward: 5′-AACCTGCTGGTGTGT GACGTTC-3′, reverse: 5′-CAGCACGAGGCTTTTTTGTTGT-3′. *IL-6* forward: 5′-ACAACCACGGCCTTCCCTACTT-3′, reverse: 5′-CACGATTTCCCAGAGAACATGTG-3′. *MCP-1* forward: 5′-CCACTCACCTGCTGCTACTCAT-3′, reverse: 5′-T GGTGATCCTCTTGTAGCTCTCC-3′. *TNF-*α forward: 5′-GC CTCTTCTCATTCCTGCTTG-3′, reverse: 5′-CTGATGAGAG GGAGGCCATT-3′. *GAPDH* forward: 5′-AACTTTGGCATT GTGGAAGG-3′, reverse: 5′-ACACATTGGGGGTAGGAACA-3′. *GAPDH* was used as an internal control.

### Statistical Analysis

All experiments have been reproduced at least three times, and all attempts at replication were successful with self-consistent results. Data represent means ± standard deviation (SD). One-way ANOVA analysis followed by a Tukey’s *post hoc* test, and Student’s *t*-test were used for statistical analysis. Statistical difference was considered as significant when *p* < 0.05.

## Results

### Astragaloside IV Prevented H9c2 Myocyte Apoptosis Induced by High Glucose/High Fat and Hypoxia

**FIGURE 1:**
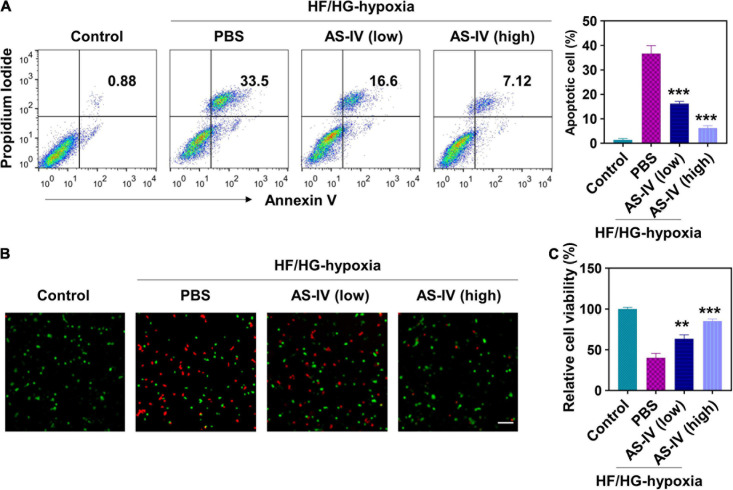
Effect of astragaloside IV (AS-IV) on high glucose/high fat and hypoxia-induced apoptosis in H9c2 myocytes. To mimic the pathological condition with high glucose and high fat (HG/HF), H9c2 myocytes were cultured in HG/HF medium for 12 h followed by hypoxia for 2 h. Then H9c2 myocytes were treated with phosphate-buffered saline (PBS) or AS-IV solution (10 or 50 ng/ml). HG/HF medium was maintained during AS-IV treatment. Cell apoptosis and proliferation were examined 48 h later. **(A)** Cell apoptosis was detected by flow cytometry after staining with propidium iodide (PI) and Annexin V-FITC. Data represent means ± standard deviation (SD). ***p < 0.001, versus PBS group, *n* = 3 biologically independent samples. **(B)** Fluorescence images of live/dead H9c2 myocytes after AS-IV treatment. Cell viability was detected using LIVE/DEAD^TM^ Viability/Cytotoxicity Kit. Live and dead cells were stained as green and red. Scale bar = 50 μm. **(C)** Cell viability was examined via Cell Count Kit-8 assay. Data represent means ± SD. **p < 0.01; ***p < 0.001, versus the PBS group, *n* = 3 biologically independent samples.

To evaluate the effects of AS-IV on apoptosis, we cultured H9c2 cells with high glucose (HG) and high fat (HF) medium for 12 h and cultured under hypoxia for 2 h. Then H9c2 cells were treated with 10 ng/ml (low dose) or 50 ng/ml (high dose) of AS-IV for 48 h. Cell apoptosis was analyzed by staining with PI and annexin V. As shown in Figure 1A, HF/HG and hypoxia treatment resulted in significantly increased PI/Annexin V double-positive cells, indicating HF/HG and hypoxia condition-induced cell apoptosis. In contrast, low- and high-dose AS-IV treatment significantly decreased the percentage of apoptotic cells in a dose-dependent manner, indicating that AS-IV prevented HF/HG and hypoxia-induced apoptosis. The preventive effects of AS-IV on cell death were further confirmed using immunofluorescence assay. After treatment, we stained H9c2 cells with LIVE/DEAD^*TM*^ Viability/Cytotoxicity Kit. As shown in Figure 1B, HF/HG and hypoxia resulted in cell death (red fluorescence reflected dead cells, green fluorescence reflected live cells), while AS-IV prevented HF/HG and hypoxia-induced cell death. Similar results were obtained using CCK-8 assay (Figure 1C). These results demonstrated that AS-IV prevented HG/HF and hypoxia-induced apoptosis in H9c2 myocytes.

### Astragaloside IV Modulated Mitogen-Activated Protein Kinase Signaling Pathway in H9c2 Cells

**FIGURE 2:**
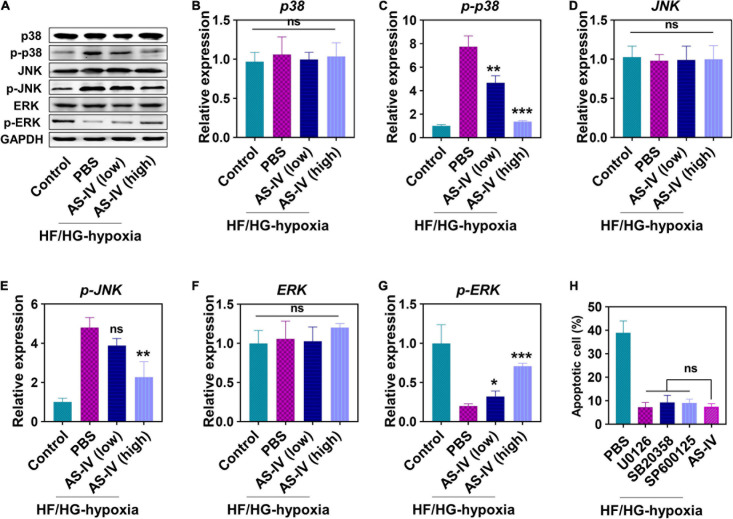
Effect of AS-IV on the protein expression in mitogen-activated protein kinase (MAPK) signaling pathway post HG/HF and hypoxia treatment. **(A)** Representative images of Western blot of MAPKs-p38 enzymes (p38), p-p38, c-Jun NH2-terminal kinases (JNK), p-JNK, extracellular signals-regulated kinases (ERK), and p-ERK. The protein expressions of p38 **(B)**, p-p38 **(C)**, JNK **(D)**, p-JNK **(E)**, ERK **(F)**, and p-ERK **(G)** were quantified by Image J software. H9c2 myocytes were cultured in HG/HF medium for 12 h followed by hypoxia for 2 h. Then H9c2 myocytes were treated with PBS, MAPK inhibitors (U0126, 25 nM; SB20358, 25 nM; SP600125, 25 nM), AS-IV (50 ng/ml), and the cell apoptosis was examined 48 h later. **(H)** Cell viability of H9c2 myocytes. Data represent means ± SD. *p < 0.05; **p < 0.01; ***p < 0.001, versus the PBS group, normalized to GAPDH level, *n* = 3 biologically independent samples.

Next, we evaluated the effects of AS-IV on MAPK signaling pathway. As shown in Figure 2A, we detected an increased amount of phospho-p38 and phospho-JNK, while a decreased amount of phospho-ERK in H9c2 cells was detected under HG/HF and hypoxia condition (Figures 2A,C,E). In contrast, AS-IV treatment decreased phospho-p38 and phospho-JNK levels but increased phospho-ERK amount in a dose-dependent manner (Figures 2A,G). AS-IV treatment did not obviously change the level of total p38, JNK, and ERK (Figures 2A,B,D,F). After quantitation, we found that both low and high doses of AS-IV significantly decreased the amount of phospho-p38. A high dose of AS-IV significantly reduced the amount of phospho-JNK. AS-IV significantly increased the amount of phospho-ERK at both low and high doses. These results indicated that AS-IV inhibited phosphorylation of p38 and JNK but promoted phosphorylation of ERK, suggesting that AS-IV regulated the activation of MAPK signaling pathway differently. Since MAPK signaling pathways were involved in the regulation of apoptosis, we further compared the effects of MAPK inhibitors including U0126 (inhibitor of MEK), SB20358 (inhibitor of p38), and SP600125 (inhibitor of JNK) on AS-IV on apoptosis. As shown in Figure 2H, these inhibitors displayed similar inhibitory effects on apoptosis when compared with AS-IV.

### Astragaloside IV Ameliorated Cardiac Function in Myocardial Infarction Diabetic Mice

**FIGURE 3:**
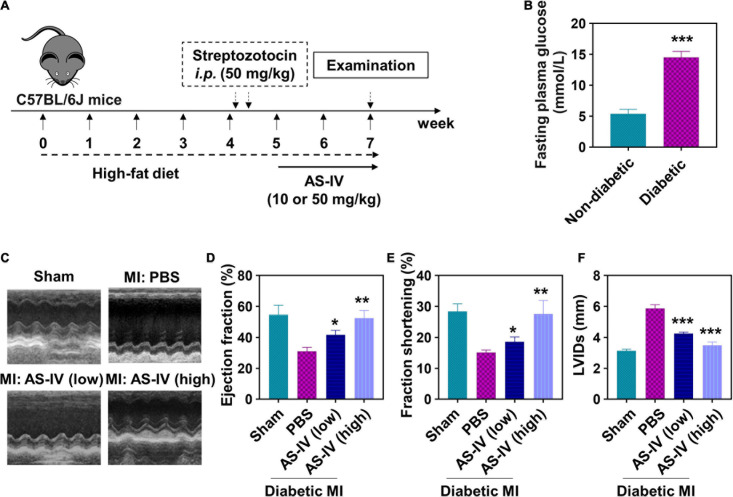
Effect of AS-IV on cardiac function in post-MI diabetic mice. **(A)** Experimental protocol. C57BL/6J mice were fed high-fat diet for 4 weeks prior to overnight fasting. Mice were then given 100 μl solution of streptozotocin (50 mg/kg body weight/day) for two consecutive days. After confirming the characteristic of diabetes mellitus, myocardial infarction models were established, and the mice were orally administrated with AS-IV (10 or 50 mg/kg/day) for 2 weeks. **(B)** Detection of fasting plasma glucose (FPG) with or without high-fat diet and streptozotocin administration. Data represent means ± SD. ***p < 0.001, *n* = 6 biologically independent mice. **(C)** Representative M-mode echocardiographic images from four respective mouse groups. Cardiac function was assessed using echocardiography at the 2 weeks post-myocardial infarction (MI). **(D)** Ejection fraction. **(E)** Fractional shortening. **(F)** LVIDs, left ventricular internal dimensions during systole (end-systolic diameter). Data represent means ± SD. *p < 0.05; **p < 0.01; ***p < 0.001, versus the PBS group, *n* = 6 biologically independent mice.

We continued to evaluate the effects of AS-IV *in vivo*. We established the diabetic mice model using streptozotocin, induced the MI in these mice, and treated them with AS-IV (Figure 3A). The diabetes in mice was confirmed by FPG (Figure 3B). Cardiac function was assessed using echocardiography. As shown in Figure 3C, we detected obvious cardiac geometry change in MI diabetic mice. MI diabetic mice treated with a high dose of AS-IV had cardiac geometry similar to control mice, indicating that AS-IV rescued the cardiac geometry in MI diabetic mice. In addition, both low and high doses of AS-IV treatment significantly enhanced ejection fraction (Figure 3D) and fraction shortening (Figure 3E), while significantly decreasing the LVIDs (Figure 3F) in MI diabetic mice. Collectively, these data indicated that AS-IV treatment improved cardiac geometry and contractile function.

### Astragaloside IV Suppressed Fibrosis and Inflammation in Myocardial Infarction Diabetic Mice

**FIGURE 4:**
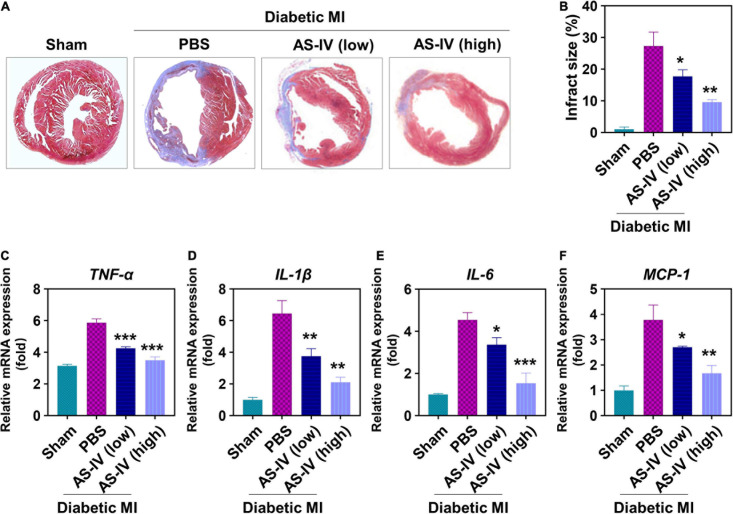
Effects of AS-IV on cardiac fibrosis and inflammation in post-MI diabetic mice. **(A)** Representative Masson’s trichrome-stained myocardial sections in hearts. Blue, scar tissue; red, viable myocardium. **(B)** Quantitation of infarct size in the four indicated experimental groups. Data represent means ± SD. **p < 0.05; **p < 0.01. *n* = 6 biologically independent mice. Quantitative analysis of mRNA expression of proinflammatory cytokines and chemokines, including TNF-α **(C)**, IL-1β **(D)**, IL-6 **(E)**, and MCP-1 **(F)**, in the border zone of infarct at 1 week post-MI. mRNA expression normalized to GAPDH expression of the same group. Data represent means ± SD. *p < 0.05; **p < 0.01; ***p < 0.001, *n* = 6 biologically independent mice.

Furthermore, we evaluated the effects of AS-IV on fibrosis and inflammation in the heart tissue of MI diabetic mice. As shown in Figure 4A, Masson’s trichrome staining illustrated a remarkable rise of fibrosis in the hearts of MI diabetic mice when compared with the heart of control mice. In contrast, AS-IV treatment decreased the fibrotic area. After quantitation, AS-IV significantly decreased infarct size in diabetic MI mice (Figure 4B). Both low and high doses of AS-IV treatments significantly decreased the mRNA expression of pro-inflammatory cytokines TNF-α (Figure 4C), IL-1β (Figure 4D), IL-6 (Figure 4E), and chemokine MCP-1 (Figure 4F). These data showed that AS-IV suppressed inflammation in diabetic MI mice.

### Astragaloside IV Regulated Mitogen-Activated Protein Kinase Signaling Pathways in Myocardial Infarction Diabetic Mice

**FIGURE 5:**
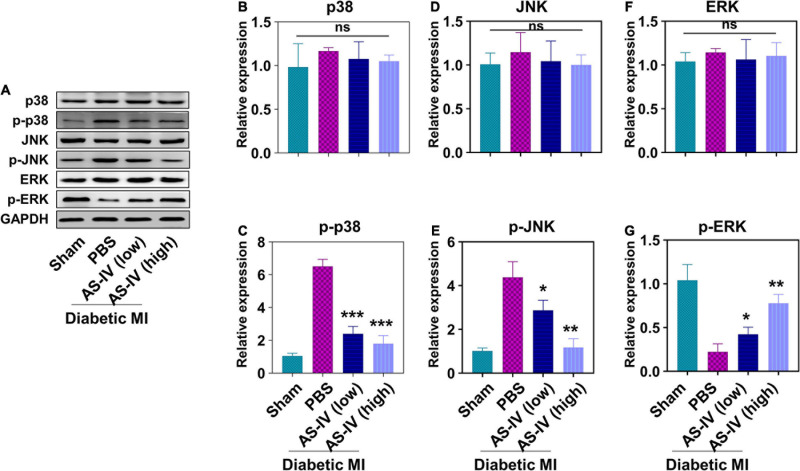
Effect of AS-IV treatment on the activation of MAPK signaling pathway in diabetic MI hearts. **(A)** Representative images of Western blot of p38, p-p38, JNK, p-JNK, ERK, and p-ERK. The protein expressions of p38 **(B)**, p-p38 **(C)**, JNK **(D)**, p-JNK **(E)**, ERK **(F)**, and p-ERK **(G)** were quantified by Image J software. Data represent means ± SD. ns, no significant difference, *p < 0.05; **p < 0.01; ***p < 0.001, versus PBS group, normalized to GAPDH level, *n* = 3 biologically independent samples.

Finally, we evaluated the effects of AS-IV on MAPK signaling pathway in the heart tissues of MI diabetic mice. We found a similar protein level of total p38 (Figures 5A,B), JNK (Figures 5A,D), and ERK (Figures 5A,F) in the heart tissue of sham mice, PBS-treated mice, and mice treated with AS-IV. We detected significantly increased amounts of phospho-p38 (Figures 5A,C) and phospho-JNK (Figures 5A,E), and decreased amount of phospho-ERK in the heart tissues of MI diabetic mice when compared with control mice. In contrast, AS-IV treatment significantly decreased the amount of phospho-p38 and phospho-JNK (Figures 5A,C), while it increased phospho-ERK (Figures 5A,G). These results demonstrated that AS-IV regulated MAPK signaling pathways in MI diabetic mice.

## Discussion

In the present study, we evaluated the effects of AS-IV on myocardial infarction. We found that AS-IV prevented HF/HG and hypoxia-induced cell apoptosis of H9c2 cells and increased the viability of these cells. AS-IV suppressed the activation of p38 and JNK signaling pathways while promoting the activation of ERK signaling pathway. In diabetic mice, AS-IV treatment ameliorated the cardiac function, prevented fibrosis, and suppressed inflammation after MI. AS-IV also differently regulated the activation of MAPK signaling pathways in diabetic MI mice. Our findings strongly suggested that AS-IV could be used as a potential therapeutic approach to treat MI in diabetes.

Myocardial infarction is the leading cause of death in patients with diabetes over the world. Increasing pieces of evidence have shown that patients with T2DM have higher risk of developing cardiovascular diseases than the risk in patients without T2DM (Sarwar et al., 2010). The molecular mechanisms of MI pathogenesis in diabetic hearts are not fully elucidated. Upregulated apoptosis of cardiomyocytes has been identified in diabetic MI rats, and the enhanced apoptosis contributed to cardiac dysfunction and fibrosis (Bäcklund et al., 2004). In the present study, we found that the HG/HF and hypoxia condition also induced apoptosis of H9c2 myocytes, while AS-IV treatment remarkably prevented HG/HF and hypoxia-induced apoptosis. AS-IV did not affect the viability of H9c2 myocytes under normal condition (data not shown), but only prevented HG/HF-induced apoptosis. Inhibition of apoptosis has been shown to protect against myocardial injury. Caspases play a pivotal role in apoptosis. Mocanu et al. (2000) found that caspase inhibitors limited myocardial infarction in MI. Chang et al. (2017) reported that the Chinese medicine Qishenkeli prevented myocardial apoptosis, improved cardiac function, decreased fibrotic area, and infarct size in heart failure rats. The anti-apoptotic activity of AS-IV has been described previously. Liu et al. (2020) reported that AS-IV prevented radiation-induced apoptosis in brain cells by suppressing the expression of pro-apoptotic proteins. In another study, Liu et al. (2017) demonstrated that AS-IV protected against IL-1β-induced chondrocyte apoptosis by upregulating autophagy activity in chondrocytes. Therefore, the anti-apoptotic activities of AS-IV widely exist, which are through multiple mechanisms.

Inflammation plays a critical role in MI. During MI, multiple cytokines are produced, and these cytokines amplify the pro-inflammatory response. For example, IL-1β is one of the major cytokines mediating the inflammatory response in MI. Inhibition of IL-1β and its signaling pathway reduced MI size and prevented LV remodeling (Abbate et al., 2008; Bujak et al., 2008). We found that AS-IV treatment results in suppressing the production of pro-inflammatory cytokines IL-1β, IL-6, TNFα, and the chemokine MCP-1 in diabetic MI mice. The anti-inflammation activities of AS-IV have been described (Zhang and Frei, 2015) reported that AS-IV inhibits the LPS-induced NF-κB activation and inflammatory cytokine expression in mice. Hsieh et al. (2020) reported that AS-IV inhibits inflammation and oxidation by inhibiting NF-κB, MAPK, and HO-1/Nrf2 signaling pathways in epithelial cells. The anti-inflammatory activities of AS-IV contribute to the amelioration of MI.

The MAPK signaling pathways participate in various cell functions and have been implicated in several diseases including MI. In experimental MI animals, ERK, JNK, and p38 MAPK signaling pathways are all activated (Yoshida et al., 2001; Ren et al., 2005). Using a dominant negative p38 transgenic mice, which express a dominant negative form of p38 MAPK, Ren et al. (2005) found that DN-p38 mice had markedly decreased cardiomyocyte apoptosis and had reduction in pathological cardiac remodeling after MI. See et al. (2004) described that the p38 inhibitor RWJ-67657 had biennial effects on cardiac remodeling and function after MI. We also demonstrated that AS-IV treatment suppressed the activation of p38 signaling pathway *in vivo* and *in vitro*. The inhibition of p38 signaling pathway should contribute to AS-IV-mediated protection against MI. Lu et al. (2020) reported that melatonin inhibited HG/HF and hypoxia-induced cardiomyocyte apoptosis by inhibiting the activation of the JNK signaling pathway, while these effects were impaired by the JNK activator anisomycin. Similar to this finding, we found that AS-IV treatment suppressed the activation of JNK and prevented HG/HF and hypoxia-induced apoptosis of H9c2 myocytes. The anti-apoptosis activity of AS-IV, which was also described in other models (Jia et al., 2014; Dong et al., 2017), should contribute to AS-IV-mediated protection against MI. The ERK signaling pathway has been reported to protect the myocardium from ischemic injury. Using Ekr heterozygous gene-targeted mice, Lips et al. described that Erk2^±^ mice had increased infarction area apoptosis. They concluded that ERK signaling protected hearts and preserved hemodynamic function after ischemia–reperfusion injury. Interestingly, in our cell model, we found that AS-IV promoted the activation of ERK signaling pathway. The upregulated ERK signaling should also contribute to AS-IV-mediated protection against MI. Therefore, AS-IV ameliorates MI by differently regulating the MAPK signaling pathways.

Several issues need to be addressed in future studies. In the current study, we only evaluated the effects of AS-IV on apoptosis and MAPK signaling pathway. As AS-IV has demonstrated other activities such as anti-oxidation, it should be interesting to evaluate the anti-oxidative effects of AS-IV in MI. In the *in vitro* experiment, we used two dosages (10 and 50 ng/ml) of AS-IV. These dosages were utilized based on results from our pilot experiments in which we used different amounts of AS-IV including 0.5, 1, 2.5, 5, 10, 15, 25, 50, 75, and 100 ng/ml. We found that 5 and 10 ng/ml exhibited similar effects on cell apoptosis, and 50, 75, and 100 ng/ml also exhibited similar effects on cell apoptosis, so 10 and 50 ng/ml were selected for further examination. In our *in vivo* model, we utilized the dosages of 10 and 50 mg/kg, which are much higher than the dosages used for intravenous administration (Zhang et al., 2006). The reason is that AS-IV had low bioavailability after oral treatment (Artursson and Karlsson, 1991; Zang et al., 2020). Therefore, it is of great importance to test other desired administration routes and amounts of AS-IV. In the present study, we identified that AS-IV regulated the activation of ERK, p38, and JNK, and it would be interesting to determine whether AS-IV affects their upstream factors MAP2Ks and MAP3Ks. It has been reported that AS-IV regulates the Akt signaling pathway (Yang et al., 2019), which is involved in apoptosis (Franke et al., 2003). Experiments need to be designed to explore the effects of AS-IV on Akt in MI.

## Conclusion

We demonstrated that AS-IV ameliorated the myocardial injury and rescued the cardiac function in MI. AS-IV prevented apoptosis and differently regulated MAPK signaling pathways in MI. These results strongly suggest that AS-IV could be used as a potential therapeutic reagent to treat MI.

## Data Availability Statement

The raw data supporting the conclusions of this article will be made available by the authors, without undue reservation.

## Ethics Statement

The animal study was reviewed and approved by Xi’an International Medical Center Hospital.

## Author Contributions

CS, GZ, TW, HR, HA, CL, JL, and LG were in charge of the data curation, data analysis, drafting of the article, and the final approval of the version to be published. WL handled the study supervision, coordination, funding support, design of the study, drafting of the article, and final approval of the version to be published. All authors contributed to the article and approved the submitted version.

## Conflict of Interest

The authors declare that the research was conducted in the absence of any commercial or financial relationships that could be construed as a potential conflict of interest.

## Publisher’s Note

All claims expressed in this article are solely those of the authors and do not necessarily represent those of their affiliated organizations, or those of the publisher, the editors and the reviewers. Any product that may be evaluated in this article, or claim that may be made by its manufacturer, is not guaranteed or endorsed by the publisher.
